# Advancements in Human Embryonic Stem Cell Research: Clinical Applications and Ethical Issues

**DOI:** 10.1007/s13770-024-00627-3

**Published:** 2024-03-19

**Authors:** Soo Jin Park, Yoon Young Kim, Ji Yeon Han, Sung Woo Kim, Hoon Kim, Seung-Yup Ku

**Affiliations:** 1 Department of Obstetrics and Gynecology, Seoul National University Hospital, Seoul, Republic of Korea; 2 Department of Obstetrics and Gynecology, Seoul National University College of Medicine, 101 Daehak-Ro Jongno-Gu, Seoul, 03080 Republic of Korea; 3 Institute of Reproductive Medicine and Population, Medical Research Center, Seoul National University, Seoul, Republic of Korea

**Keywords:** Human embryonic stem cells, Regenerative medicine, Clinical trials, Ethics, Medical, Stem cell research

## Abstract

**Background:**

The development and use of human embryonic stem cells (hESCs) in regenerative medicine have been revolutionary, offering significant advancements in treating various diseases. These pluripotent cells, derived from early human embryos, are central to modern biomedical research. However, their application is mired in ethical and regulatory complexities related to the use of human embryos.

**Method:**

This review utilized key databases such as ClinicalTrials.gov, EU Clinical Trials Register, PubMed, and Google Scholar to gather recent clinical trials and studies involving hESCs. The focus was on their clinical application in regenerative medicine, emphasizing clinical trials and research directly involving hESCs.

**Results:**

Preclinical studies and clinical trials in various areas like ophthalmology, neurology, endocrinology, and reproductive medicine have demonstrated the versatility of hESCs in regenerative medicine. These studies underscore the potential of hESCs in treating a wide array of conditions. However, the field faces ethical and regulatory challenges, with significant variations in policies and perspectives across different countries.

**Conclusion:**

The potential of hESCs in regenerative medicine is immense, offering new avenues for treating previously incurable diseases. However, navigating the ethical, legal, and regulatory landscapes is crucial for the continued advancement and responsible application of hESC research in the medical field. Considering both scientific potential and ethical implications, a balanced approach is essential for successfully integrating hESCs into clinical practice.

## Introduction

The field of stem cell research has undergone a significant transformation with the advent of human embryonic stem cells (hESCs). Since their pioneering isolation in 1998, hESCs have been at the forefront of scientific inquiry due to their unique ability for self-renewal and pluripotency [1, 2]. This comprehensive review article delves into the advancements, challenges, and ethical considerations surrounding hESCs and their implications for regenerative medicine.

Over the past two decades, the potential of hESCs to revolutionize the treatment of various diseases has been increasingly recognized [3, 4]. Their capacity to differentiate into diverse cell types offers promising prospects for repairing or replacing damaged tissues, especially in conditions where current treatments are limited [5–8]. However, the journey of hESC research is not without its complexities. Ethical considerations regarding the use of human embryos have sparked intense debates and have had a profound impact on public perception and the regulatory framework governing hESC research [9, 10].

The therapeutic applications of hESCs encompass both systemic and localized approaches, including intravenous or intramuscular injections and surgical implantation, sometimes combined with bioscaffolds [11]. These strategies are broadly classified into transient dosing for temporary therapeutic effects and permanent implantation for long-term tissue repair and regeneration [12, 13]. Despite these advancements, challenges in ensuring consistency in hESC properties across different experimental settings continue to pose hurdles in translating laboratory findings into clinical therapies [14, 15].

While induced pluripotent stem cells (iPSCs) have emerged as an alternative, hESCs still hold distinct advantages, particularly in the understanding of genetic diseases and human development [16, 17]. Despite the ethical complexities and slower pace of clinical research compared to iPSCs, hESCs remain a crucial tool in biomedical research [18, 19]. Their unique position in providing insights into early human development and genetic disorders underscores their invaluable role in medical science [17].

This review aims to provide an in-depth analysis of the current state of clinical trials involving hESCs, emphasizing their role in regenerative medicine. We explore the evolving landscape of hESC research, highlighting the need for ongoing scientific exploration, ethical deliberation, and regulatory guidance to fully realize the therapeutic potential of hESCs in improving patient care and advancing medical science.

## Methodology

This narrative review was conducted to assess the clinical applications of hESCs. The primary aim was to gather and analyze data from various sources to understand the current state and advancements in hESC research.

For database search, we utilized ClinicalTrials.gov (https://clinicaltrials.gov/) and EU Clinical Trials Register (https://www.clinicaltrialsregister.eu/) for identifying ongoing and completed clinical trials involving hESCs. Also, we used PubMed and Google Scholar to retrieve published clinical trial reports and peer-reviewed articles on hESCs. Studies and trials were included based on their focus on the clinical application of hESCs. Those not directly involving hESCs or outside the scope of clinical application were excluded. The review primarily targeted articles and trials published or conducted in the last five years to maintain contemporary relevance.

For data extraction and analysis, key information extracted included the study title, indication, participant number, study site, study period, study design, and NCT number. This data was organized systematically to provide a clear overview of the current trends and progress in the field of hESC research in clinical applications.

### Overview of clinical trials in hESC research

**Fig. 1:**
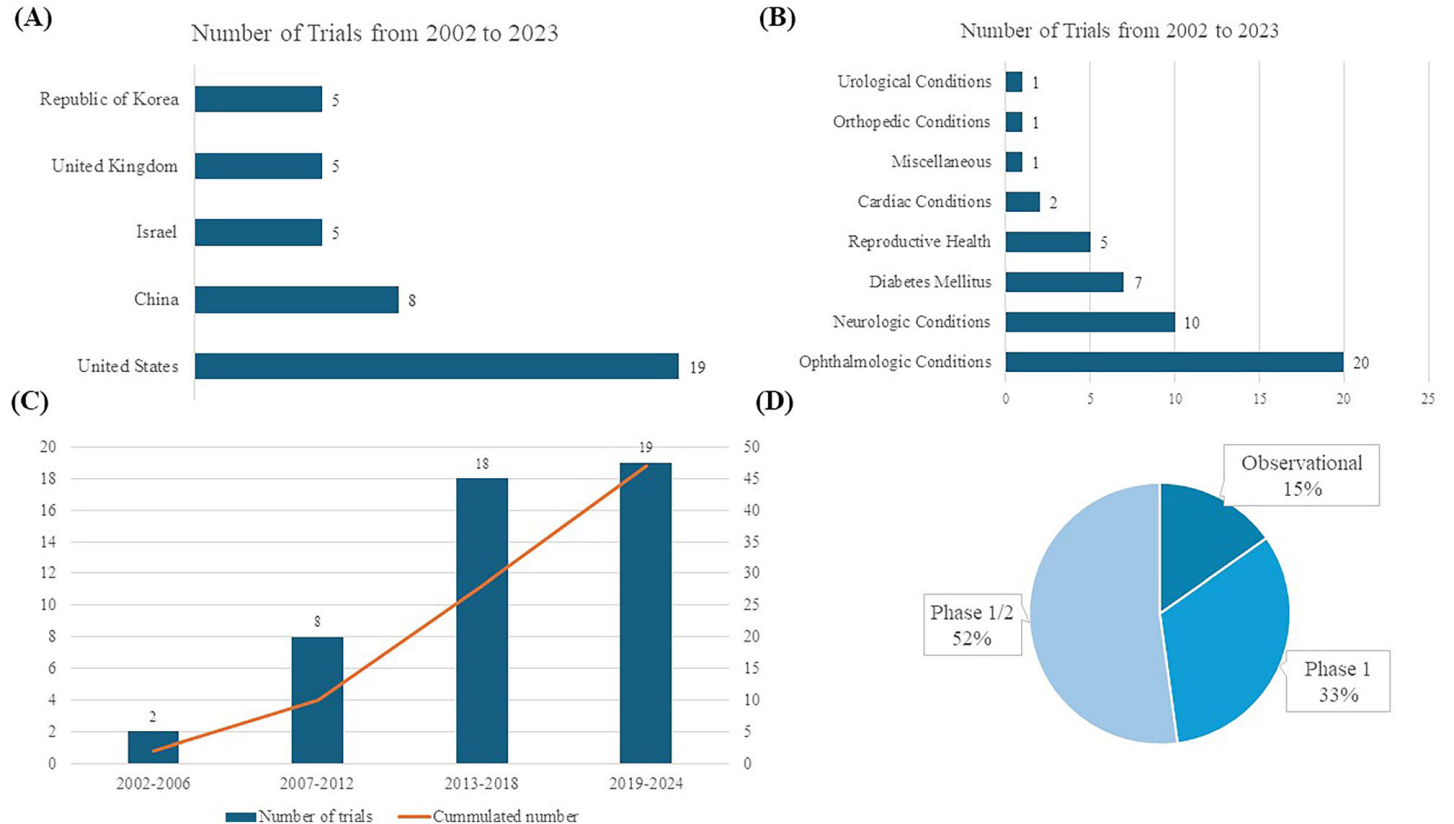
Numbers of trials on human embryonic stem cells (**A**) Global Geographical Distribution of Human Embryonic Stem Cell Clinical Trials (**B**) Distribution of Trials by Disease Category (**C**) Frequency of Trials Across Specific Diseases (**D**) Distribution of Clinical Trials Across Different Phases

Figure 1 displays key aspects of hESC clinical trials included in this review. The first clinical trial registration was in 2002, and the largest number of registered trials were in the United States (19, 40.4%), followed by China (8, 17.0%; Fig. 1A). By disease category, the largest number of trials were related to ophthalmologic conditions (20, 42.6%), followed by neurologic conditions (10, 21.3%), and clinical studies were mainly conducted on diabetes mellitus (7, 14.9%; Fig. 1B). Figure 1C shows the number of trial registrations and the cumulative number of clinical studies by year. There has been a sharp increase since 2012. (Fig. 1C), and by study design, phase 1 or phase 1/2 designs predominate, accounting for 88% (Fig. 1D). When looking at studies by a specific disease, dry age-related macular degeneration (AMD) is the most common with 8 (18.2%), followed by type 1 diabetes mellitus (T1DM, 7, 15.9%) and Stargardt Macular Dystrophy (SMD, 5, 11.4%).

### Disease-specific analysis

#### Ophthalmologic diseases

Retinal degeneration is a significant ophthalmologic disease that affects the eye and vision, including dry AMD, SMD, wet AMD, retinitis pigmentosa (RP), diabetic retinopathy, and myopic macular degeneration, among others [20–22]. These conditions often lead to severe vision impairment or blindness. Traditional treatments primarily focus on slowing the progression of these diseases but generally fall short of providing substantial visual improvement. For instance, while laser therapy is beneficial in the early stages, there is no established treatment for late-stage dry AMD [23]. In cases of wet AMD, therapies such as anti-VEGF can be administered through intravitreal infusion (e.g., ranibizumab, bevacizumab, aflibercept, and brolucizumab), yet this disease requires continuous treatment and monitoring due to its chronic nature [24–27]. Stem cell therapy, particularly involving retinal pigment epithelium (RPE) degeneration, has emerged as a promising approach in eye diseases [28]. The RPE is vital for maintaining photoreceptor health and is tasked with recycling photopigments and clearing shed photoreceptor segments [29]. hESCs have shown significant potential in rescuing photoreceptors and enhancing vision in preclinical macular degeneration models [30]. One of the initial forays into stem cell therapy using hESCs was directed at treating dry AMD using hESC-derived RPE. Several key factors contributed to this early focus on retinal conditions. Primarily, the unique immune privilege of the eye, reinforced by the blood-ocular barrier, significantly lowers the risk of rejection of transplanted cells—a crucial aspect in the success of any stem cell-based therapy [31, 32]. Moreover, the eye's transparency permits the non-invasive tracking of the introduced cells through methods like optical coherence tomography or microperimetry, enabling continuous monitoring and evaluation of the therapy's effectiveness [33]. The eye's distinct and isolated structure also minimizes the spread of these cells to other body parts, thereby reducing the likelihood of unintended systemic effects [34]. Furthermore, the absence of synaptic layers in retinal cells aids in their smoother integration [29]. Lastly, the irreversible progression of many retinal disorders and the absence of adequate existing treatments have necessitated the development of innovative therapeutic strategies, thereby placing retinal ailments at the forefront of hESC research and application.

Dry AMD, a prevalent and progressive ophthalmologic disease affecting elderly patients, is characterized by the degeneration of the RPE layer and impairment of central vision [21]. The pivotal role of RPE in the pathophysiology of dry AMD makes it a prime target for therapeutic interventions. The potential of stem cells, especially hESCs, in this context, lies in their ability to differentiate into RPE cells, thereby offering the possibility of replacing damaged or degenerated RPE with healthy, functional cells. Preclinical studies in animal models and *in vitro* experiments have provided substantial evidence supporting the role of stem cells, including hESCs, in treating dry AMD [35–37].

For example, in Yucatan minipigs, a preclinical study assessed CPCB-RPE1, a hESC-derived retinal pigment epithelium monolayer [35]. The study successfully placed CPCB-RPE1 implants in the subretinal space without breakage, and histological analysis confirmed the survival of hESC-RPE cells as an intact monolayer for one month [35]. Another study used differentiated hESC-RPE replacement therapy on albino rabbit eyes induced with NaIO3, employing a 25-gauge transvitreal pars plana vitrectomy (PPV) technique [36]. Xeno-free hESC-RPE monolayer on a polyester substrate survived and retained functionality for up to four weeks with short-term immunosuppression in a rabbit dry AMD model [37]. These studies demonstrate the feasibility of generating RPE cells from stem cells and their potential to integrate into the retina, potentially restoring RPE function and rescuing photoreceptors. Also, the critical advantage of hESC-RPE is their reduced risk of uncontrolled proliferation, as they are fully differentiated.

Clinical trials have been conducted to test the safety and feasibility of hESC-derived RPE for dry AMD, as outlined in Table 1. Dry AMD has been the subject of the most significant number of clinical trials, with studies dating back to 2011 (Table 1). The first study involved MA09-hRPE (NCT01344993; NCT01674829; NCT02122159), derived from the MA09 hESC line, a xenograft product with ex vivo exposure to mouse embryonic cells [38]. Produced by isolating RPE patches when embryoid body formation was confirmed, this treatment was tested in three different dose cohorts (50,000, 100,000, and 150,000 cells) for patients with dry AMD and SMD [39]. Encouragingly, the study revealed no signs of adverse events like cell proliferation or immune rejection. In addition, the best-corrected visual acuity improved in 10 eyes, and measures related to vision-related quality of life showed enhancements [39]. In a clinical trial of MA09-hESC-derived RPE cells conducted with an Asian population, which included four participants, there was no evidence of adverse proliferation or tumorigenesis [40]. Furthermore, one patient experienced improved visual acuity, while the remaining three maintained stable visual acuity throughout the trial [40]. In the USA, a phase 1/2 clinical study was conducted using CPCB-RPE1, a composite implant consisting of a synthetic parylene substrate and a polarized monolayer of adherent hESC-RPE cells (NCT02590692). This study demonstrated safety and tolerability in legally blind patients with dry AMD [41, 42]. However, graft survival remains a significant challenge, influenced by factors like aging of Bruch's membrane, subretinal scarring, para-inflammation, and choroid ischemia [33].

**Table 1:** Registered trials of human embryonic stem cells for ophthalmologic disease

NCT number	Disease	Study title	Product	Enrollment	Start date	Study status	Phases	Study site
NCT01691261	Acute Wet Age-Related Macular Degeneration	A Study Of Implantation Of Retinal Pigment Epithelium In Subjects With Acute Wet Age Related Macular Degeneration	PF-052096388	10	14-Oct-21	Recruiting	Phase 1	United Kingdom
NCT02463344	Dry Age-Related macular degeneration	Long Term Follow Up of Sub-retinal Transplantation of hESC Derived RPE Cells in Patients With AMD	MA09-hRPE	11	25-Feb-13	Completed	Observational	United States
NCT03305029	Dry Age-Related macular degeneration	The Safety and Tolerability of Sub-retinal Transplantation of SCNT-hES-RPE Cells in Patients With Advanced Dry AMD	SCNT-hES-RPE Cells	3	1-May-16	Unknown	Phase 1	Republic of Korea
NCT01344993	Dry Age-Related macular degeneration	Safety and Tolerability of Sub-retinal Transplantation of hESC Derived RPE (MA09-hRPE) Cells in Patients With Advanced Dry Age Related Macular Degeneration	MA09-hRPE	13	9-Jun-11	Completed	Phase 1/2	United States
NCT01674829	Dry Age-Related macular degeneration	A Phase I/IIa, Open-Label, Single-Center, Prospective Study to Determine the Safety and Tolerability of Sub-retinal Transplantation of Human Embryonic Stem Cell Derived Retinal Pigmented Epithelial(MA09-hRPE) Cells in Patients With Advanced Dry Age-related Macular Degeneration(AMD)	MA09-hRPE	12	1-Sep-12	Unknown	Phase 1/2	Republic of Korea
NCT02286089	Dry Age-Related macular degeneration	Safety and Efficacy Study of OpRegen for Treatment of Advanced Dry-Form Age-Related Macular Degeneration	OpRegen	24	1-Apr-15	Competed	Phase 1/2	Israel
NCT02590692	Dry Age-Related macular degeneration	Study of Subretinal Implantation of Human Embryonic Stem Cell-Derived RPE Cells in Advanced Dry AMD	CPCB-RPE1	16	16-Feb-16	Unknown	Phase 1/2	United States
NCT03046407	Dry Age-Related macular degeneration	Treatment of Dry Age Related Macular Degeneration Disease With Retinal Pigment Epithelium Derived From Human Embryonic Stem Cells	hESC-RPE	10	6-Sep-17	Unknown	Phase 1/2	China
NCT02755428	Dry Age-Related macular degeneration	Subretinal Transplantation of Retinal Pigment Epitheliums in Treatment of Age-related Macular Degeneration Diseases	hESC-RPE	10	1-Jan-18	Unknown	Phase 1/2	China
NCT02749734	Macular Degeneration	Clinical Study of Subretinal Transplantation of Human Embryo Stem Cell Derived Retinal Pigment Epitheliums in Treatment of Macular Degeneration Diseases	Q-CTS-hESC-2-RPE	15	1-May-15	Unknown	Phase 1/2	China
NCT02903576	Macular Degeneration	Stem Cell Therapy for Outer Retinal Degenerations	hESC-RPE	15	1-Aug-15	Completed	Phase 1/2	Brazil
NCT03167203	Macular Degeneration	A Safety Surveillance Study in Subjects With Macular Degenerative Disease Treated With Human Embryonic Stem Cell-derived Retinal Pigment Epithelial Cell Therapy	hESC-RPE	36	8-Jan-18	Recruitment	Phase 1/2	United Kingdom
NCT02122159	Myopic Macular Degeneration	Research With Retinal Cells Derived From Stem Cells for Myopic Macular Degeneration	MA09-hRPE	0	1-Mar-13	Withdrawn	Phase 1/2	United States
NCT03944239	Retinitis Pigmentosa	Safety and Efficacy of Subretinal Transplantation of Clinical Human Embryonic Stem Cell Derived Retinal Pigment Epitheliums in Treatment of Retinitis Pigmentosa	hESC-RPE	10	1-May-20	Unknown	Phase 1	China
NCT03963154	Retinitis pigmentosa	Interventional Study of Implantation of hESC-derived RPE in Patients With RP Due to Monogenic Mutation	hESC-RPE	7	19-Aug-19	Not yet recruiting	Phase 1/2	France
NCT02445612	Stargardt macular dystrophy	Long Term Follow Up of Sub-retinal Transplantation of hESC Derived RPE Cells in Stargardt Macular Dystrophy Patients	MA09-hRPE	13	11-Jul-12	Completed	Observational	United States
NCT02941991	Stargardt macular dystrophy	A Follow up Study to Determine the Safety and Tolerability of Sub-retinal Transplantation of Human Embryonic Stem Cell Derived Retinal Pigmented Epithelial (hESC-RPE) Cells in Patients With Stargardt's Macular Dystrophy (SMD)	hESC-RPE	12	16-Jan-13	Completed	Observational	United Kingdom
NCT01625559	Stargardt macular dystrophy	Safety and Tolerability of MA09-hRPE Cells in Patients With Stargardt's Macular Dystrophy(SMD)	MA09-hRPE	3	1-Sep-12	Unknown	Phase 1	Republic of Korea
NCT01345006	Stargardt macular dystrophy	Sub-retinal Transplantation of hESC Derived RPE(MA09-hRPE)Cells in Patients With Stargardt's Macular Dystrophy	MA09-hRPE	13	16-Jun-11	Completed	Phase 1/2	United States
NCT01469832	Stargardt macular dystrophy	Safety and Tolerability of Sub-retinal Transplantation of Human Embryonic Stem Cell Derived Retinal Pigmented Epithelial (hESC-RPE) Cells in Patients With Stargardt's Macular Dystrophy (SMD)	MA09-hRPE	12	13-Dec-11	Completed	Phase 1/2	United Kingdom

AMD: Age-Related Macular Degeneration; ESC: Embryonic Stem Cell; hESC-RPE: Human Embryonic Stem Cell-Derived Retinal Pigment Epithelium; NCT Number: National Clinical Trial Number; RP: Retinitis Pigmentosa; RPE: Retinal Pigment Epithelium; SMD: Stargardt's Macular Dystrophy

SMD, a prevalent retinal dystrophy affecting young individuals, is characterized by progressive vision loss, primarily caused by mutations in the ABCA4 gene, which leads to dysfunction of the ABCR protein expressed in retinal photoreceptors [43]. Currently, there are no established treatments to effectively improve vision in SMD, similar to the situation in dry AMD. Promising outcomes have been observed in preclinical models, including the safe subretinal injection of retinal pigment epithelium (RPE) derived from hESC. This approach was tested in a phase 1 clinical trial in the USA (NCT02941991). The WA-099 hESC line demonstrated the ability to spontaneously differentiate into RPE cells, with subsequent isolation of pigmentation cells. A suspension of these hESC-derived RPE cells, containing 1.0 × 10^6 cells in 0.1 mL, was surgically implanted subretinally in all eyes using a pars plana vitrectomy (PPV) approach [44]. The study's findings indicated no adverse events during the one-year postoperative follow-up period. Additionally, the treated eyes had no significant improvement in visual acuity [44]. In China, researchers Li et al. evaluated the Q-CTS-hESC-2 cell line-derived RPE in a 5-year follow-up study on seven patients and reported no significant adverse reactions and some temporary improvements in visual function, though two patients showed a long-term decrease in vision (NCT02749734) [45]. Sung et al., from the Republic of Korea, reported a 3-year study on Asian patients, also finding no serious adverse events and reporting stable or improved BCVA in some patients (NCT01625559) [46].

RP is a group of inherited retinal disorders characterized by the progression of vision loss due to photoreceptor degeneration, affecting approximately 1 in 4,000 individuals worldwide [47, 48]. A Phase 1/2 clinical trial of RP with monogenic mutations is ongoing (NCT03963154), with interim analysis showing no adverse events in seven patients [49]. While these studies confirm the long-term safety and tolerability of hESC-RPE cell transplantation, they also highlight the need for further research to improve efficacy, including better patient selection and treatment methodologies, as significant and consistent improvements in visual function are yet to be established.

#### Neurologic diseases

The utilization of stem cell therapy derived from hESCs in treating neurological disorders is an emerging and promising area of research. As illustrated in Fig. 1B, neurologic diseases are among the most researched applications in this field. This branch of medical science addresses a diverse spectrum of neurological conditions, including Parkinson's disease (PD), amyotrophic lateral sclerosis (ALS), spinal cord injuries (SCI), and multiple sclerosis. These disorders present considerable treatment challenges, largely due to the complexity of the nervous system and the typically permanent nature of neuronal damage involved. Ongoing studies are displayed in Table 2.

**Table 2:** Registered trials of human embryonic stem cells for neurologic disease

NCT number	Disease	Study title	Product	Enrollment	Start date	Study status	Phases	Study site
NCT03482050	Amyotrophic lateral Sclerosis	A Study to Evaluate Transplantation of Astrocytes Derived From Human Embryonic Stem Cells, in Patients With Amyotrophic Lateral Sclerosis (ALS)	AstroRx®	16	12-Apr-18	Completed	Phase 1/2	Israel
NCT05135091	Epilepsy	FIH Study of NRTX-1001 Neural Cell Therapy in Drug-Resistant Unilateral Mesial Temporal Lobe Epilepsy	NRTX-1001	40	16-Jun-22	Recruiting	Phase 1/2	United States
NCT04956744	Multiple Sclerosis	A Study to Evaluate the Safety, Tolerability, and Exploratory Efficacy of IMS001 in Subjects With Multiple Sclerosis	IMS001	30	31-Aug-21	Recruiting	Phase 1	United States
NCT04802733	Parkinson's Disease	Phase 1 Safety and Tolerability Study of MSK-DA01 Cell Therapy for Advanced Parkinson's Disease	MSK-DA01	12	3-May-21	Not yet recruiting	Phase 1	United States
NCT05635409	Parkinson's Disease	A Trial to Determine the Safety and Tolerability of Transplanted Stem Cell Derived Dopamine Neurons to the Brains of Individuals With Parkinson's Disease	STEM-PD	8	30-Nov-22	Recruiting	Phase 1	United Kingdom
NCT03119636	Parkinson's Disease	Safety and Efficacy Study of Human ESC-derived Neural Precursor Cells in the Treatment of Parkinson's Disease	hESC-NPC	50	1-May-17	Unknown	Phase 1/2	China
NCT01217008	Spinal Cord Injury	Safety Study of GRNOPC1 in Spinal Cord Injury	LCTOPC1	5	1-Oct-10	Competed	Phase 1	United States
NCT02302157	Spinal Cord Injury	Dose Escalation Study of AST-OPC1 in Spinal Cord Injury	LCTOPC1	25	1-Dec-18	Competed	Phase 1/2	United States
NCT04812431	Spinal Cord Injury	Safety and Exploratory Efficacy of Transplantation Therapy Using PSA-NCAM( +) NPC in AIS-A Level of Sub-acute SCI	PSA-NCAM( +) NPC	5	23-Sep-21	Recruiting	Phase 1/2	Republic of Korea
NCT04631406	Stroke	A Safety and Tolerability Study of Neural Stem Cells (NR1) in Subjects With Chronic Ischemic Subcortical Stroke (ISS)	NR1	30	4-Jan-21	Recruiting	Phase 1/2	United States

ALS: Amyotrophic Lateral Sclerosis; ESC: Embryonic Stem Cell; hESC-NPC: Human Embryonic Stem Cell-Derived Neural Precursor Cells; NCT Number: National Clinical Trial Number; PSA-NCAM( +): Polysialylated Neural Cell Adhesion Molecule Positive Neural Precursor Cells; SCI: Spinal Cord Injury

The first-in-patient clinical trial on neurologic disease was conducted on SCI patients [50]. Oligodendrocyte progenitor cells (LCTOPC1), which are also nomenclature as AST-OPC1 or GRNOPC1, is the world's first hESC-derived therapy, and the phase 1 trial was approved by US-FDA in 2009, and the first patient was enrolled in 2011 (NCT01217008) [50, 51]. Recent 10-year follow-up study results on five participants who received intraparenchymal injections of LCTOPC1 showed no serious adverse effects during follow-up, with 80% of patients showing MRI evidence of tissue matrix formation at the injury site [51]. This pivotal study, leading to a subsequent cervical dose escalation trial (NCT02302157), demonstrated the safety of hESC-derived therapies using LCTOPC1. In the trial, 25 participants with C4-7 spinal injuries received a single dose of 2, 10, or 20 million LCTOPC1 cells and low-dose tacrolimus for 60 days [52]. Despite some adverse events, including 29 serious ones, the treatment was well tolerated, with MRI scans showing no significant complications, and at a 1-year follow-up, 96% of participants improved by at least one level of neurological function, and 32% improved by two or more levels [52].

Additionally, research has shown that neural precursor cells marked by polysialic acid-neural cell adhesion molecule (PSA-NCAM), derived from hESC, can enhance neural tissue integrity in a rat stroke model [53]. Building on these findings, a phase 1/2a clinical trial (NCT04812431) is currently underway to assess the safety and efficacy of PSA-NCAM( +)-NPC for patients with sub-acute C4-C7 level spinal cord injuries. In this trial, the cells will be delivered intrathecally across five sites, and participants will be monitored for one year and five months as part of a follow-up study.

PD is a neurodegenerative disease characterized primarily by the loss of dopaminergic neurons in the substantia nigra, a region of the brain integral to controlling body movement. This loss leads to the classic symptoms of PD, including tremors, rigidity, bradykinesia, and postural instability [54]. The potential of hESC-based therapies in PD lies in their ability to differentiate into dopaminergic neurons, the type of cell lost in the disease [55]. The goal of transplanting hESC-derived cells in PD treatment is to replace the depleted neurons and normalize dopamine levels in the brain, which could help alleviate PD symptoms. MSK-DA01, a midbrain dopamine neuron cell derived from hESCs, is currently undergoing a Phase 1 trial in the United States (NCT04802733). A preclinical study on MSK-DA01 demonstrated successful graft survival and improved behavior in rats with 6-hydroxydopamine-induced lesions, a model for PD. Importantly, these studies revealed no adverse effects related to the graft cells and no unexpected cell proliferation outside the brain, indicating a promising safety profile for this innovative therapy [56].

STEM-PD, another product consisting of dopaminergic neuronal progenitor cells derived from hESCs, has also been evaluated in a preclinical study [57]. This study showed the precise stereotactic injection of STEM-PD into a pig model and demonstrated effective innervation of the targeted brain regions. Additionally, this intervention led to a reversal of motor deficits in the pig model of Parkinson's disease, demonstrating the potential efficacy of STEM-PD in addressing the symptoms associated with this neurodegenerative disorder [57]. Presently, STEM-PD is the subject of a phase 1 clinical trial in the United Kingdom, which is in the process of recruiting eight patients, and this trial marks a significant step in evaluating the safety and potential efficacy of STEM-PD in human subjects, specifically targeting the treatment of PD (NCT05635409).

A research team in China successfully derived dopaminergic neurons from hESCs and demonstrated sustained behavioral improvements over two years in a monkey model of PD [58]. This significant advancement in stem cell research has led to the registration of a Phase 1 clinical trial (NCT03119636). However, the current status of this trial remains unknown.

ALS, a severe neurodegenerative condition, is characterized by the deterioration of both upper and lower motor neurons (MNs), resulting in the progressive paralysis of muscles controlled by these neurons [59]. While FDA-approved treatments like riluzole have demonstrated some efficacy in prolonging survival, there remains a significant unmet need for more effective ALS therapies [60]. Recent evidence points to the involvement of astrocytes in the pathogenesis of ALS [61]. AstroRx®, a novel cell therapy derived from hESCs, has shown promise in addressing this gap, as evidenced by the outcomes of its recent Phase 1/2a clinical trial [62]. AstroRx®, administered as a single intrathecal injection, was tested in two cohorts of ALS patients—a low-dose and a high-dose group, each consisting of five patients (NCT03482050). The administration of AstroRx® showed a clinically significant impact lasting for three months post-treatment, with particularly notable effects observed in a group of rapid progressors [62].

NR1, an hESC-derived neural stem cell, is under investigation for chronic ischemic stroke patients who are 6–60 months post-ischemic subcortical mid-cerebral artery stroke (NCT04631406). Six patients underwent transplantation with NR1, and there was a notable improvement in the Mugl-Meyer motor score. Additionally, all six patients exhibited a transient flair signal that resolved within two months, which correlated with neurological recovery [63].

#### Diabetes mellitus

Type 1 Diabetes Mellitus (T1DM) commonly manifests in childhood and adolescence and is marked by a chronic autoimmune condition leading to the loss of insulin-producing beta cells in the pancreas [64]. Unlike Type 2 DM, which often relates to lifestyle and insulin resistance, T1DM is primarily driven by an autoimmune response [64]. In stem cell therapy for T1DM, two main strategies have emerged: one involves replacing the missing insulin-producing beta cells, while the other focuses on immunomodulation to safeguard existing beta cells from further autoimmune destruction [65]. Seven registered clinical trials for stem cell-based treatment of T1DM using hESC are summarized in Table 3.

**Table 3:** Registered trials of human embryonic stem cells for diabetes mellitus

NCT number	Disease	Study title	Product	Enrollment	Start date	Study status	Phases	Study site
NCT02939118	T1DM	One-Year Follow-up Safety Study in Subjects Previously Implanted With VC-01	VC-01™ Combination Product	51	1-Mar-24	Not yet recruiting	Observational	United States
NCT05210530	T1DM	An Open-Label, FIH Study Evaluating the Safety and Tolerability of VCTX210A Combination Product in Subjects With T1D	VCTX210A	7	24-Jan-22	Completed	Phase 1	Canada
NCT02239354	T1DM	A Safety, Tolerability, and Efficacy Study of VC-01 Combination Product in Subjects With Type I Diabetes Mellitus	VC-01™ Combination Product	19	1-Sep-14	Completed	Phase 1/2	United States
NCT03163511	T1DM	A Safety, Tolerability, and Efficacy Study of VC-02™ Combination Product in Subjects With Type 1 Diabetes Mellitus and Hypoglycemia Unawareness	VC-02™ Combination Product	49	6-Jul-17	Completed	Phase 1/2	United States
NCT04678557	T1DM	A Study to Evaluate Safety, Engraftment, and Efficacy of VC-01 in Subjects With T1 Diabetes Mellitus	VC-01™ Combination Product	31	25-Jun-19	Completed	Phase 1/2	United States
NCT04786262	T1DM	A Safety, Tolerability, and Efficacy Study of VX-880 in Participants With Type 1 Diabetes	VX-880	17	29-Mar-21	Recruiting	Phase 1/2	United States
NCT04678557	T1DM	A Study to Evaluate Safety, Engraftment, and Efficacy of VC-01 in Subjects With T1 Diabetes Mellitus (VC01-103)	VC-01™ Combination Product	31	12/11/2021	Terminated	Phase 1/2	United States

ESC: Embryonic Stem Cell; FIH: First-In-Human; NCT Number: National Clinical Trial Number; T1DM: Type 1 Diabetes Mellitus

Schulz and colleagues described the creation of the VC-01 composite product utilizing pancreatic endoderm cells (PEC-01) obtained from CyT49 hESCs with a retrievable semi-permeable encapsulating device drug delivery system [66]. VC-02, developed in 2017, is an advanced model featuring multiple large pores across the membrane to facilitate vascularization while maintaining immune isolation [67]. VC-01 was investigated in phase 1/2 trial (NCT02239354; NCT04678557; NCT02939118) and VC-02 was investigated in phase 1/2 trial (NCT03163511). In the phase 1/2 study of the VC-01 product, immunosuppressants were not administered, leading to a host reaction against the implant, ultimately resulting in its destruction, and the study was terminated [68]. A Phase 1/2 study involving 17 patients with T1DM was carried out following a modification in the VC-02 device. This study demonstrated successful engraftment and insulin release in 63% of the cases, and as early as six months post-implantation, 35.3% of the participants showed positive C-peptide levels. These results indicate the potential of VC-02 as a viable alternative for T1DM treatment. However, it's important to note that some reported adverse events were primarily related to the surgical procedures of implanting or explanting the device and the side effects of immunosuppression therapy [69]. VCTX210A represents an innovative approach that uses pancreatic endodermal cells (PEC210A) derived from hESC. These cells have been genetically modified using the CRISPR/Cas9 (Clustered Regularly Interspaced Short Palindromic Repeats/CRISPR-associated protein 9) technology. This modification enhances the cells' survival against the patient's immune system, thereby addressing the challenge of graft versus host disease [70]. Additionally, VX880, a fully differentiated pancreatic islet cell product derived from hESC designed to treat T1DM, is undergoing clinical investigation (NCT04786262). Interim data analysis from this study has yielded positive results, indicating that the treatment successfully restored insulin production in the first two patients enrolled in the trial [71].

#### Female reproductive organ and genitourinary disease

The field of female reproductive organ disorders is increasingly looking towards stem cell therapy and cutting-edge biomedical technologies for potential treatments, as shown in Table 4. Intravenous injection of hESC-derived mesenchymal cells (hESC-MCs) showed restoration of ovarian function induced by the chemotherapeutic agent in a murine model [72, 73]. A product, hESC-MC, has been explored by a Chinese research group for treating moderate to severe intrauterine adhesion (NCT04232592). Additionally, a therapy involving hESC-MC product is currently being investigated as a potential treatment for primary ovarian insufficiency (NCT03877471). Additionally, Table 5 showcases the application of hESC-derived mesenchymal stem cell therapy, specifically MR-MVC-01, which is currently under investigation for treating interstitial cystitis, as per the clinical trial registered under NCT04610359.

**Table 4:** Registered trials of human embryonic stem cells for female reproductive organ

NCT number	Disease	Study title	Product	Enrollment	Start date	Study status	Phases	Study site
NCT02713854	Infertility/Subfertility	BAP-EB as a Predictive Tool for Endometrial Receptivity and Pregnancy Outcome of IVF Treatment	BAP-EB	240	1-Feb-17	Completed	NA	China, Hong Kong
NCT04232592	Intrauterine Adhesions	Clinical Safety Study of Human Embryonic Stem Cell Derived Mesenchymal Cells in the Treatment of Moderate and Severe Intrauterine Adhesions	hESC-MC	32	1-Jan-20	Unknown	Phase 1	China
NCT03877471	Ovarian Insufficiency	Mesenchymal Stem Cells (MSCs)—Like Cell Transplantation in Women With Primary Ovarian Insufficiency	hESC-MSC like cell	28	3-Apr-19	Unknown	Phase 1	China

BAP-EB: Blastocyst Attachment to a Prepared Endometrium—Embryonic Bodies; ESC: Embryonic Stem Cell; hESC-MC: Human Embryonic Stem Cell-Derived Mesenchymal Cells; hESC-MSC: Human Embryonic Stem Cell-Derived Mesenchymal Stem Cells; IVF: *In Vitro* Fertilization; MSCs: Mesenchymal Stem Cells; NCT Number: National Clinical Trial Number; NA: Not Applicable

**Table 5:** Registered trials of human embryonic stem cells for cardiac, urological disease and miscellaneous topics

NCT number	Disease	Study title	Product	Enrollment	Start date	Study status	Phases	Study site
NCT02057900	Heart Failure	Transplantation of Human Embryonic Stem Cell-derived Progenitors in Severe Heart Failure	hESC-derived-CD15 + Isl-1 + progenitors	10	27-May-13	Completed	Phase 1	France
NCT05068674	Ischemic Heart Disease	Human Embryonic Stem Cell-Derived Cardiomyocyte Therapy for Chronic Ischemic Left Ventricular Dysfunction	hESC-cardiomyocyte	18	22-Mar-22	Recruiting	Phase 1	United States
NCT04610359	Interstitial Cystitis	Safety of Human Embryonic Stem Cell (hESC)-Derived Mesenchymal Stem Cells in Interstitial Cystitis	MR-MC-01	3	20-Oct-20	Unknown	Phase 1	Republic of Korea
NCT03839238	Meniscus Injury	Safety Observation on hESC Derived MSC Like Cell for the Meniscus Injury	hESC-MSC like cell	18	4-Jan-19	Unknown	Phase 1	China
NCT00353197	NA	Derivation of New Human Embryonic Stem Cell Lines Lines for Clinical Use	hESC lines	80	7-Jul-02	Recruiting	Observational	Israel
NCT00353210	NA	The Derivation of Human Embryonic Stem Cell Lines From PGD Embryos	hESC lines derived from embryos diagnosed as abnormal by PGD testing	70	6-Apr-04	Recruiting	Observational	Israel
NCT01165918	NA	Derivation of New Human Embryonic Stem Cell Lines: Identification of Instructive Factors for Germ Cells Development	hESC lines	50	1-Oct-10	Unknown	Observational	Israel

ESC: Embryonic Stem Cell; hESC: Human Embryonic Stem Cell; hESC-cardiomyocyte: Human Embryonic Stem Cell-Derived Cardiomyocytes; hESC-derived-CD15 + Isl-1 + progenitors: Human Embryonic Stem Cell-Derived CD15 + Isl-1 + Progenitor Cells; hESC-MSC: Human Embryonic Stem Cell-Derived Mesenchymal Stem Cells; MSC: Mesenchymal Stem Cell; NCT Number: National Clinical Trial Number; PGD: Preimplantation Genetic Diagnosis

#### Cardiovascular disease

In the field of heart failure treatment, the innovative application of human embryonic stem cells (hESCs) offers a promising alternative to conventional therapies. Table 5 also highlights hESC-derived cardiac progenitor cell-based products in treating heart failure and ischemic heart disease, as illustrated in the clinical trials registered under NCT02057900 and NCT05068674. The ESCORT trial (NCT02057900), conducted in France, marked a pioneering venture in employing hESC-derived cardiomyocytes for heart failure treatment, setting a precedent that has been followed by the HECTOR trial (NCT05068674) in the United States, initiated in 2022. The ESCORT trial, focusing on patients with severe ischemic left ventricular dysfunction, demonstrated the feasibility and safety of using hESC-derived cardiovascular progenitor cells, embedded in a fibrin patch, applied to the damaged heart areas during coronary artery bypass surgery [74]. The results, including the production of a highly purified batch of progenitor cells and significant symptomatic improvements in patients, though with instances of silent alloimmunization, have laid the groundwork for future explorations in this domain. The HECTOR trial in the U.S. is building upon this foundation with a novel approach, utilizing hESC-derived cardiomyocytes (hESC-CMs) to enhance survival and cardiac function in patients with chronic left ventricular dysfunction secondary to myocardial infarction. This phase I dose-escalation pilot study is designed as an initial safety assessment to determine the maximum tolerated dose (MTD) before proceeding to a phase II randomized, double-blinded, placebo-controlled study. Approximately eighteen patients who are scheduled for cardiac catheterization and meet all inclusion/exclusion criteria will participate in this initial phase. The HECTOR trial represents a significant step forward in the application of hESC-CMs in cardiac therapy, with great anticipation for its potential to revolutionize the treatment of heart failure and related conditions.

### Challenges and ethical considerations

As we explore the burgeoning field of hESC research and its clinical applications, it becomes crucial to examine the accompanying ethical and practical challenges thoroughly. While this area of research offers groundbreaking possibilities in treating various diseases, it is intertwined with complex ethical, legal, and social issues, particularly due to the involvement of human embryos.

#### Derivation of hESC

In the field of hESC research, the ethical implications surrounding the derivation of these cells from embryos are paramount. hESCs are typically harvested from embryos at the blastocyst stage approximately 5–6 days post-fertilization. This stage of development is critical because it leads to the inevitable destruction of the embryo, a primary ethical concern in this field of research [19, 75–77].

Due to their pluripotency, the significant potential of hESCs makes them a valuable asset in understanding disease mechanisms, drug testing, and potential regenerative therapies [78]. Moreover, hESCs are obtained early in induced pluripotent development, making them crucial for studying human developmental processes and various diseases [17]. They play a vital role, especially when embryos are discarded after positive preimplantation genetic testing (PGT) results, contributing to our understanding of genetic abnormalities and disease ecology [17].

Regarding the moral status of the embryo, there are varying views. The Catholic perspective often sees life beginning at fertilization, while Judaism and Islam view the blastocyst as having the potential for life but not as fully alive [79, 80]. Hinduism and Buddhism do not provide a clear doctrinal definition of life's beginning, adopting a more philosophical and spiritual perspective [81].

The use of surplus IVF embryos in hESC research is often defended under the principle of proportionality. This approach favors using them for stem cell research due to the broader potential benefits compared to enhancing IVF techniques [17]. The utilization of embryos with monogenic defects (PGT-M) or aneuploidies (PGT-A) for deriving disease-specific stem cells is seen as a promising avenue for advancing the understanding of specific diseases and developing targeted treatments [9, 17].

In summary, hESC research presents a complex ethical landscape. The scientific and medical benefits of hESCs must be balanced against the moral considerations surrounding the use of human embryos, necessitating a nuanced approach to this rapidly evolving field.

#### Regulatory issues

In the realm of research involving hESCs, regulatory issues play a crucial role, varying significantly across different countries. Obtaining approval from institutional review boards (IRBs) and adhering to regulations set by authoritative bodies are pivotal steps in developing and progressing hESC-related research and development.

Procedures involving the transfer of stem cells are subject to specific regulations. This encompasses the process of transferring stem cell materials, which requires careful adherence to legal and ethical guidelines [15, 82]. It's essential to ensure that the transfer agreements are comprehensive, detailing any restrictions and obligations related to using the materials and associated data [83, 84]. Such transfers must respect donor rights and comply with the regulatory frameworks of both the donating and receiving entities.

The process of creating stem cell products that are safe for clinical use involves several critical steps. This includes extensive testing for genetic stability and absence of contaminants, ensuring the cells' identity and functionality, and verifying that they meet the stringent safety standards required for clinical application [82]. These procedures are designed to safeguard patient safety and ensure the efficacy of the stem cell products.

Overall, the development and research involving hESCs must navigate a complex landscape of regulatory requirements. These regulations are in place to ensure the ethical use of human stem cells, the protection of donor rights, and the safety and efficacy of stem cell-based therapies. Compliance with these regulations is not only a legal requirement but also a cornerstone in maintaining the integrity and credibility of stem cell research.

## Conclusion

The exploration of hESCs over the past two decades has opened new frontiers in medical science, particularly in the fields of regenerative medicine and cell-based therapies. The landmark discovery and subsequent developments have brought immense potential for understanding and treating a wide range of diseases, from genetic disorders to degenerative conditions.

However, the journey of hESC research is intertwined with a plethora of ethical, legal, and regulatory challenges. The ethical considerations, primarily regarding the use of human embryos, highlight the delicate balance between scientific advancement and moral imperatives. Different religious and cultural perspectives on embryo status underline this debate's complexity. As we have seen, approaches to this issue vary significantly worldwide, influencing the regulatory landscape and research in different countries.

The advancements in hESC research also underscore the importance of robust regulatory frameworks and adherence to ethical standards. From acquiring embryonic materials to developing stem cell-based products for clinical use, each step requires careful consideration of ethical guidelines, safety standards, and regulatory compliance. The involvement of IRBs and adherence to international standards and guidelines are critical in ensuring that the research is conducted responsibly and with the utmost respect for human life and dignity.

Looking ahead, the field of hESC research holds immense promise. With continued technological advancements and a deeper understanding of stem cells' capabilities, we stand on the brink of revolutionary medical breakthroughs. However, the path forward must be navigated with a commitment to ethical principles, regulatory compliance, and public engagement. By upholding these standards, the scientific community can ensure that the benefits of hESC research are realized in a manner that respects human values and contributes positively to human health and well-being.

In conclusion, hESC research represents scientific innovation, ethical reflection, and regulatory prudence. As we continue to advance in this field, it is imperative to maintain a balanced approach that fosters scientific discovery while honoring ethical obligations and regulatory requirements. The future of hESC research, promising as it is, depends on our collective ability to navigate these complex and multifaceted challenges.

## Data Availability

The datasets generated during and/or analysed during the current study are available from the corresponding author on reasonable request.
